# Providing Nursing Home Care in an Era of Increasing Scarcity: The Case of Pennsylvania

**DOI:** 10.1093/geroni/igab046.1935

**Published:** 2021-12-17

**Authors:** Edward Miller, Molly Wylie, Elizabeth Simpson, Marc Cohen

**Affiliations:** 1 University of Massachusetts Boston, Boston, Massachusetts, United States; 2 University of Massachusetts Boston, University of Massachusetts boston, Massachusetts, United States; 3 University of Massachusetts Boston, University of Massachusetts Boston, Massachusetts, United States

## Abstract

Medicaid financing of nursing home (NH) care provides the strongest safety net for low income older adults, persons who have high-intensity long-term care (LTC) needs, and consumers with exorbitant LTC costs. Yet, NHs currently face serious threats to their financial viability, particularly in the context of the COVID-19 pandemic, where the costs of caring for residents in a safe way have increased significantly, even as the ability to recoup these costs from the Medicaid program has been constrained. The purpose of this study is to assess key demand and supply factors affecting the performance of the NH industry in Pennsylvania over time. It draws from several large, national data sources, including NH Compare, LTCFocus.org, the U.S. Bureaus of the Census and Labor Statistics, and Certification and Survey Provider Enhanced Reports, as well as state-level population projections and Departments of Health and Human Services data. An aggregate database was constructed with historical data points at the facility, regional, and state level. Annual total and regional trends were examined from 2010 to 2020. Findings suggest a growing gap between what NHs require to meet the needs of residents and the level of reimbursement paid by the largest funder: Medicaid. Considering demographic trends, this gap will only grow over time in the absence of policy change. The pandemic has further highlighted the existing challenges resulting from an underfunded service infrastructure and the need for additional investment if NHs are to provide high quality care to a growing cohort of older adults requiring support.

